# Restoring Anomalous Water Surface in DOM Product of UAV Remote Sensing Using Local Image Replacement

**DOI:** 10.3390/s25134225

**Published:** 2025-07-07

**Authors:** Chunjie Wang, Ti Zhang, Liang Tao, Jiayuan Lin

**Affiliations:** 1 Chongqing Jinfo Mountain Karst Ecosystem National Observation and Research Station, School of Geographical Sciences, Southwest University, Chongqing 400715, China; xndxdlkx3321563794@email.swu.edu.cn; 2 Engineering Research Center of South Upland Agriculture (Ministry of Education), College of Agronomy and Biotechnology, Southwest University, Chongqing 400715, China; zhangti@swu.edu.cn; 3 School of Computer Science, Sichuan University Jinjiang College, Chengdu 620860, China

**Keywords:** unmanned aerial vehicle (UAV), digital orthophoto map (DOM), multisource seed filling, contour finding, affine transformations, image replacement

## Abstract

In the production of a digital orthophoto map (DOM) from unmanned aerial vehicle (UAV)-acquired overlapping images, some anomalies such as texture stretching or data holes frequently occur in water areas due to the lack of significant textural features. These anomalies seriously affect the visual quality and data integrity of the resulting DOMs. In this study, we attempted to eliminate the water surface anomalies in an example DOM via replacing the entire water area with an intact one that was clipped out from one single UAV image. The water surface scope and boundary in the image was first precisely achieved using the multisource seed filling algorithm and contour-finding algorithm. Next, the tie points were selected from the boundaries of the normal and anomalous water surfaces, and employed to realize their spatial alignment using affine plane coordinate transformation. Finally, the normal water surface was overlaid onto the DOM to replace the corresponding anomalous water surface. The restored water area had good visual effect in terms of spectral consistency, and the texture transition with the surrounding environment was also sufficiently natural. According to the standard deviations and mean values of RGB pixels, the quality of the restored DOM was greatly improved in comparison with the original one. These demonstrated that the proposed method had a sound performance in restoring abnormal water surfaces in a DOM, especially for scenarios where the water surface area is relatively small and can be contained in a single UAV image.

## 1. Introduction

UAV remote sensing technologies have been increasingly applied in the fields of resources and environment due to their advantages of high resolution, flexible takeoff and landing, and low cost [1]. Among them, colorful images with a certain degree of overlap acquired by UAV optical remote sensing are processed by aerial triangulation and orthorectification to produce the principal data products, including point clouds, digital surface models (DSMs), and digital orthophoto maps (DOMs). During the process of aerial triangulation, one of the key steps is to seek tie points from adjacent images [2]. The water surface, as a common feature type, is characterized by relatively homogeneous image texture, which makes the matching of feature points extremely difficult. As a result, some anomalies such as data holes, texture distortion, and texture stretching were frequently observed in the water surface portion of the final DOM product [3], which seriously affected the visual quality and data integrity.

At present, research on improving the quality of UAV-acquired images mainly focuses on removing some abnormal elements in the images, such as shadows, water interference, and anomalous texture. Liu et al. [4] proposed a texture-preserving local color transfer method for shadow compensation in UAV images, which constructs pixel-level correspondence between illuminated and shadowed regions based on spatial proximity and color similarity. Alvarado-Robles et al. [5] suggested a color-transfer algorithm based on the CIE Lab and HSV color spaces, which effectively corrected image colors and removed shadows caused by uneven lighting. Wang et al. [6] extracted abnormal components from RGB images based on highlight detection, and used the Fast Marching Method (FMM) to repair abnormal water regions with specular reflection. Yao et al. [7] proposed an unsupervised model called DewaterGAN, which employed a physics-based attention module to effectively remove water interference from images while preserving authenticity. Lin et al. [8] designed an auto-adaptive filter based on the similar frequency phenomena in the gray-scale histogram and the Fourier spectrum to effectively remove the stripe noise in the NDVI image. The methods in these studies mainly involved color transfer, spectrum transformation, a physical mechanism, and so on. However, there were few studies on eliminating the anomalies that occur in the process of aerial triangulation.

In practical applications, researchers attempted to apply an adjacent texture replacement method to restore the abnormal water surface in the DOM [9]. The core idea of this method was to use the existing texture information in the image to fill or replace the missing or anomalous parts. Firstly, the subareas with anomalies in the water surface were manually identified. Then, an image patch nearby with normal texture was copied to repeatedly overlay onto the anomalous areas, achieving the goal of eliminating water surface anomalies. Finally, the edge pixels of the restored areas were blended with the surrounding pixels to achieve a natural transition. Although this method was regarded as intuitive in operation, some problems were encountered in practical applications, especially in terms of texture consistency, operation efficiency, and restoration quality [10].

Another potential method is the functionality provided by some aerial triangulation software (e.g., Acute3D ContextCapture v4.4.10) [11], which can be used to eliminate anomalies in data products of UAV remote sensing such as point clouds and DSMs [12]. The correction of point clouds and DSMs is mainly based on geometric information, whereas DOM restoration requires the handling of texture and color information. Therefore, this functionality is not as effective for DOMs. In addition, similar functionality was generally not provided by other aerial triangulation software including Pix4Dmapper 4.5.6 and Agisoft Metashape Pro v1.5.5 [13]. Hence, it is typically necessary for users to manually eliminate water surface anomalies occurring in DOM products.

In this study, taking a pond and the neighborhood of Southwest University, China, as the study area, we captured the UAV images and obtained a DOM with anomalies such as data holes and texture stretching in the pond. After careful observation, it was found that the water portion of the original UAV-acquired image generally did not contain anomalies, but was affected by other problems such as geometric distortion and partial coverage of the water surface. Therefore, from the original UAV-acquired imagery, one single image, which contained the entire water surface and had relatively small geometric distortion, was selected to restore the anomalous water surface. The major objectives include (1) automatically extracting the water surface scope and boundary in the single image using the multisource seed filling algorithm and contour-finding algorithm; (2) spatially aligning the normal and anomalous water surfaces using the affine transformation; and (3) replacing the anomalous water surface in the DOM with the normal one to achieve the purpose of eliminating water surface anomalies.

## 2. Study Area and Data

### 2.1. Study Area and DOM

**Figure 1:**
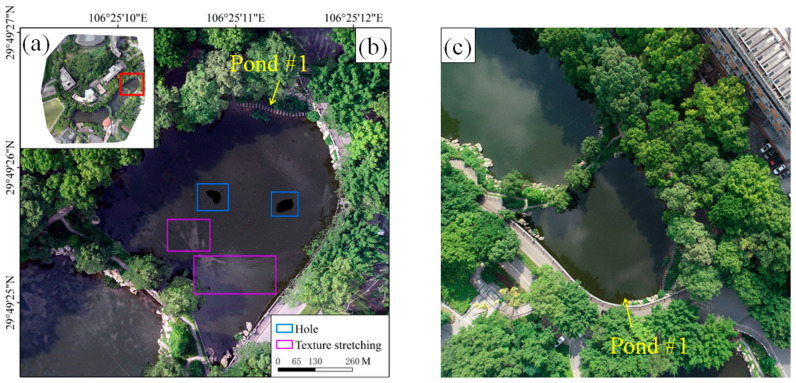
(**a**) DOM of the study area produced from UAV-captured images; (**b**) Pond #1 with anomalous water surface in DOM; and (**c**) one single image containing the entire water surface of Pond #1.

The study area was located in the campus of Southwest University, which was situated in the center of Beibei District in Chongqing, China. The environment of Southwest University is natural, with a large number of planted tall trees, and is known as the “Forest University”. There are also some small ponds scattered between trees and buildings. In this study, the target of the UAV operation is the Chongde Ponds and the surrounding area on campus. The DJI Phantom 4 Professional was used for image collection. It was equipped with a SONY FC6310 camera featuring a focal length of 8.8 mm, resulting in a high-resolution image of 5472 × 3648 pixels. The following parameters were set for the aerial photography operation: a relative flight altitude of 120 m, a heading overlap rate of 85%, and a side overlap rate of 70%. A total of 47 UAV images with a spatial resolution of 3.47 cm were captured over the time period from 11:20 to 11:50 a.m. under clear sky conditions on 3 June 2024. The raw overlapping images were processed using Pix4Dmapper 4.5.6, and the steps including aerial triangulation, feature point matching, orthorectification, and image mosaicking were sequentially conducted, eventually producing the DOM of the study area (seen in Figure 1a). It had a spatial reference defined by the WGS84 geodetic coordinate system and UTM projection/Zone 48.

### 2.2. Single Image Selection

As indicated in Figure 1b, obvious anomalies including data holes and texture stretching were observed in the water surface of Pond #1 of Chongde Ponds, whose total area was approximately 0.68 hm^2^. From the original UAV-acquired imagery, one single image containing the entire water surface of Pond #1 was picked out (Figure 1c). In addition, a small geometric deformation was another criterion for image selection, which was satisfied by the image shown in Figure 1c.

## 3. Methodology

**Figure 2:**
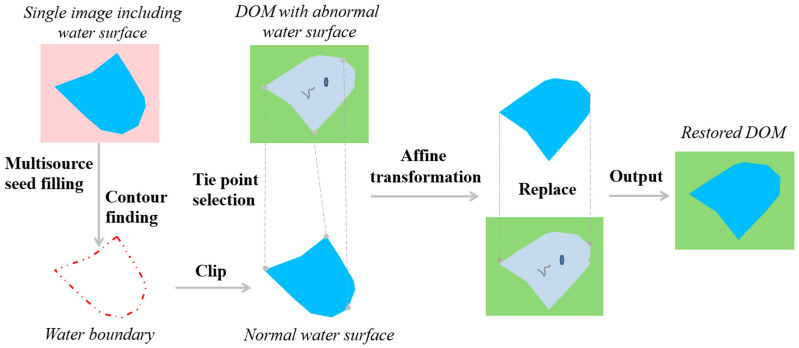
Flowchart of restoring an anomalous water surface in a DOM product of UAV remote sensing using image replacement. DOM is short for digital orthophoto map.

The process for restoring anomalous water surface areas in DOMs proposed in this study is illustrated in Figure 2. First of all, the multisource seed filling algorithm is utilized to automatically obtain the entire water surface scope in the single UAV-acquired image and the boundary is extracted using the contour-finding algorithm. The boundary is then used to clip out the image portion of intact water surface. Secondly, multiple pairs of feature points are selected from the boundaries of the normal and abnormal water surfaces as the tie points, which are used to realize their spatial alignment using the affine plane coordinate transformation. Finally, the spatially aligned normal water surface is overlaid onto the DOM to replace the corresponding anomalous water surface, outputting the restored DOM product.

### 3.1. Boundary Extraction of Normal Water Surface

#### 3.1.1. Water Surface Zoning

In this study, it is a prerequisite for restoring an abnormal water surface in DOM to accurately extract the boundary of the normal water surface. In drone-acquired optical imagery, water surfaces typically exhibit distinctive visual and color characteristics. The overall color and texture of the water surface differ significantly from the surrounding terrestrial areas. Meanwhile, the interior of the water surface also exhibits characteristics such as continuity and homogeneity, which provide essential foundations for the precise extraction of the water surface boundary.

**Figure 3:**
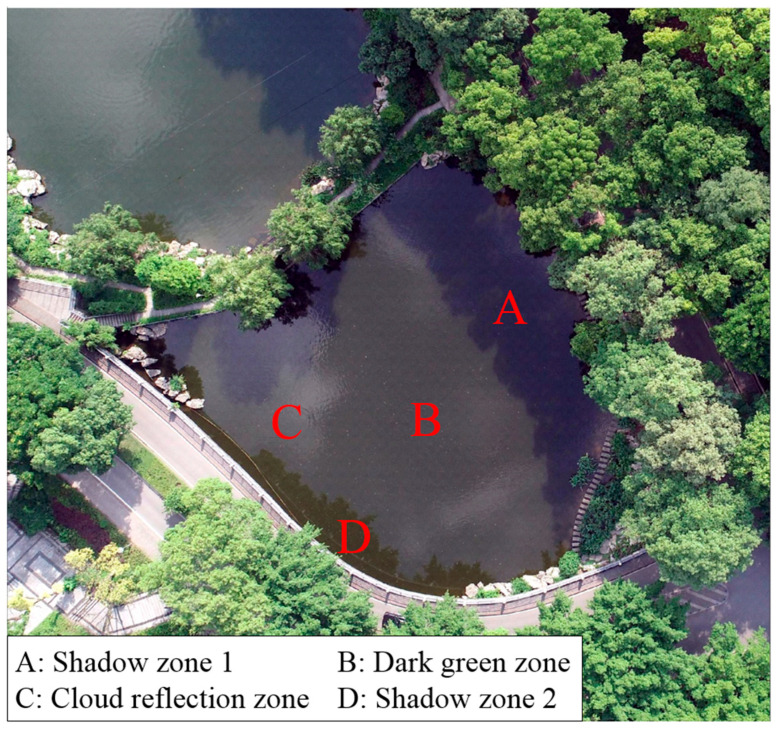
Water surface zoning of Pond #1.

However, there are zonal differences in the Pond #1 water surface of the selected single image, which can be specifically divided into four characteristic zones (seen in Figure 3): shadow zone 1 (A) and 2 (D), dark green water zone (B), and white cloud reflection zone (C). Each type of zone exhibits distinct color and texture characteristics. These zones were formed by multiple environmental factors, including lighting conditions, cloud cover, shore vegetation, and image capture angle. For example, the water surface zone directly exposed to sunlight appears brighter, revealing the original green color of the pond, while those shaded by trees on the shore exhibit dark tone. Additionally, the white clouds reflected at specific locations of the water surface introduce additional color and texture variations, causing the upper-left corner of the image to appear with a white hue. Therefore, these water surface zones pose challenges for automatically extracting the water surface boundary of Pond #1.

#### 3.1.2. Water Surface Acquisition with Multisource Seed Filling

According to the color continuity within each zone of the water surface, we utilize the seed filling algorithm [14] as a basis to obtain the complete scope of the water surface. The algorithm initially selects the seed point in the target zone and searches for adjacent pixels based on the color or attributes of the seed point. If the color or attribute of adjacent pixels is the same as the seed point, the pixel is filled and its neighboring pixels will be evaluated recursively until boundary pixels are encountered, and the filling process stops. In the practical application of this algorithm, colors or attributes that are completely consistent with the seed point appear too strict. In this study, the CIE76 color difference algorithm [15] is adopted to quantitatively assess the color differences between neighboring pixels and seed points. In addition, due to the presence of multiple color zones on the target water surface, the single seed-filling algorithm is not very suitable for obtaining the entire water surface scope. Therefore, we propose an improved multisource seed-filling algorithm to achieve this objective, which effectively addresses the inherent limitations of traditional methods. For example, the traditional scanline algorithm [16] has an obvious efficiency bottleneck when dealing with complex concave polygons, and its computational complexity rises significantly with the increase in the number of polygon vertices. On the other hand, although the traditional seed-filling algorithm is able to deal with regions of complex shapes, it shows obvious deficiencies when facing regions with color heterogeneity. It is difficult to accurately identify and distinguish regions with similar spatial features but different color attributes. The proposed algorithm not only improves the processing efficiency of complex geometric shapes, but also enhances the recognition ability of color heterogeneity by introducing the multisource seed point collaboration mechanism and the color similarity assessment based on CIE76 color difference. Therefore, it provides a better solution for filling complex regions in images.

The CIE76 color difference calculation is based on the LAB color space, which evaluates pixel similarity through color differences. The LAB color space, proposed by the International Commission on Illumination (CIE) in 1976 [17], is a device-independent color model designed to align with human visual perception. It comprises three components: the luminance component (L), the green-red component (A), and the blue-yellow component (B). Unlike the RGB color space, the LAB color space uniformly represents visual color differences and is widely used in color correction and image analysis. The core principle of the CIE76 algorithm is to quantify color differences using the Euclidean distance in the LAB color space. In this study, each neighboring pixel is compared with the fixed seed point by calculating their Euclidean distance (∆*E*_ab_) in the LAB color space. RGB values are necessarily converted into LAB coordinates. If the distance between a neighboring pixel and the seed point is below the preset color difference threshold, the pixel is deemed part of the water surface zone and is filled. The calculation formula is as follows:

(1) $$\Delta E_{ab}=\sqrt{(L_{1}-L_{2}{)}^{2}+(a_{1}-a_{2}{)}^{2}+(b_{1}-b_{2}{)}^{2}}$$

where *L*_1_ and *L*_2_ represent the luminance components of the two colors; *a*_1_ and *a*_2_ denote the green-red components, while *b*_1_ and *b*_2_ are the blue-yellow components. The Euclidean distance ∆*E*_ab_ between the two coordinates serves as the quantitative measurement of color difference.

**Figure 4:**
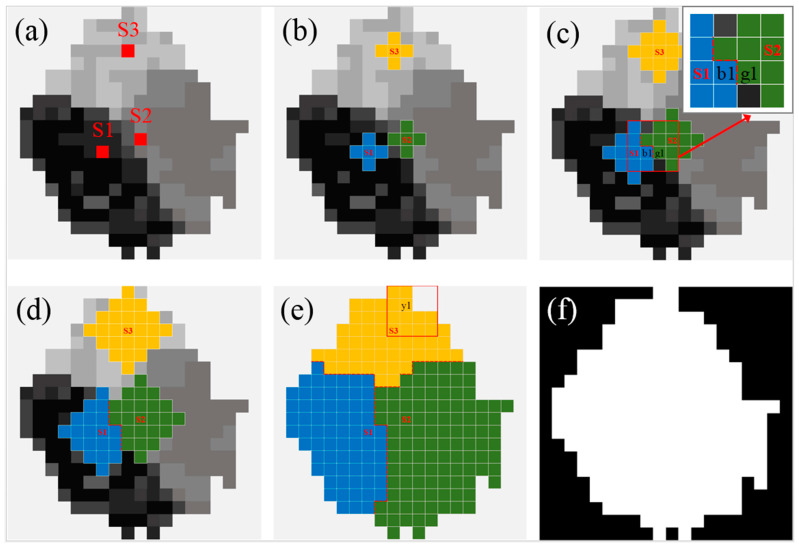
Automatic acquisition of water surface scope using the multisource seed-filling algorithm. (**a**) Fixed seed points in each color zone; (**b**) the first round of synchronous filling from seed points; (**c**) delineating the border between adjacent filling zones; (**d**) an intermediate state during the filling process; (**e**) the final synchronous filling results; and (**f**) the binary image of the merged entire water surface.

As illustrated in Figure 4, the multisource seed-filling algorithm performs a synchronous filling process by selecting multiple independent fixed seed points in each color zone (S1–S3 in Figure 4a). All of the seed points synchronously evaluate the filling feasibility of adjacent pixels according to the threshold of CIE76 color difference (Figure 4b). This process repeats until all sufficiently similar pixels are included in the zone (Figure 4c–e). Finally, the filled extents of all zones can be merged to achieve the entire scope of the water surface (Figure 4f). This multisource collaboration mechanism not only retains the advantage of the recursive processing of the seed-filling algorithm, but also effectively improves the filling efficiency of multiple color zones through parallel processing.

The key is that all seeds share the global traversal records during the synchronous filling process. Before any neighboring pixel is filled, its filling status will be first checked from the global traversal records. In this way, the border conflicts of adjacent zones can be reasonably treated. As indicated in Figure 4c, b1 and g1 are two neighboring pixels. Pixel g1 is first filled by seed S2, and marked as traversed. Pixel b1 is first filled by seed S1, and also marked as traversed. When b1 performs the neighbor judgment, it will skip g1 according to the traversal records, even if g1 meets the color difference threshold. Similarly, g1 will skip b1. This effectively avoids filling the same pixel for multiple times, improving the filling efficiency. In Figure 4e, the right neighboring pixel of y1 is not filled because the color difference between them is less than the specified threshold. This constitutes the outer boundary of the water surface.

In the specific implementation of filling, the algorithm first pushes all of the neighboring pixel points of the seed point into the stack, and pops one pixel point from the stack each time. Then, the color difference between that point and its seed point is calculated. If the distance is less than a specified threshold, the point is filled and its unvisited neighboring pixels are also pushed into the stack. This process is repeated until the stack is empty, i.e., all eligible pixels are effectively filled. The first in last out (FILO) mechanism of the stack ensures that the algorithm can gradually traverse the entire water surface, guaranteeing the spatial continuity and filling completeness. At the same time, it can flexibly cope with the complex structure of the image to ensure that all eligible pixels are filled accurately.

#### 3.1.3. Water Boundary Extraction with Contour Finding

After the entire water surface is obtained using the multisource seed-filling algorithm, the extraction of the water boundary becomes the next important task, which usually includes steps of image binarization, contour finding, and water boundary identification.

**Figure 5:**
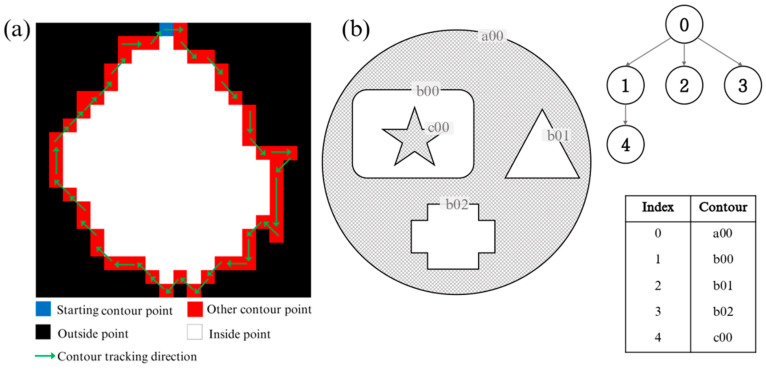
(**a**) Contour point finding in a binary image; (**b**) contour index and hierarchy.

As shown in Figure 4f, the filled water surface and the rest of the parts are binarized. Then, the FindContours function in the OpenCV library [18,19] is used to find contours in the binary image. It operates by identifying continuous edge transitions between black and white pixels. As illustrated in Figure 5a, the contour finding initiates from a starting point, and progressively tracks adjacent edge points until a closed loop (contour) is achieved.

For each contour, not only the coordinates of the pixels that make up it are obtained, but also a tuple recording its relative relationship in the contour hierarchy. The tuple has four elements [20]: next contour index, previous contour index, first child contour index, and parent contour index. As illustrated in Figure 5b, contour b00 has an index of 1. It is enclosed by contour a00 (parent contour), but contains contour c00 (child). Therefore, the tuple of contour b00 is represented as (2, 0, 4, 0). The water surface boundary is the outermost contour, that is, the contour without a parent in the contour hierarchy. Therefore, the water boundary can be conveniently achieved by detecting whether the parent contour in the tuple of all contours is null (0) or not. As it organizes complex geometric relationships into a navigable tree structure, the hierarchical representation enables sophisticated contour analysis while maintaining computational efficiency. This approach allows the rapid isolation of the target boundary from potentially numerous found contours in high-resolution imagery.

#### 3.1.4. Color Difference Threshold Selection

In Section 3.1.2, the color difference threshold is a key parameter for the multisource seed-filling algorithm, and is defined as the maximum allowable grayscale value of the color difference between adjacent pixels and local seed points. In this section, a systematic evaluation and selection mechanism is established to determine the optimal color difference threshold.

First of all, a representative threshold sequence should be set in advance according to experiences and experiments. For each candidate threshold, the identical seed points in all zones are used to perform the multisource seed filling operation, and the entire filled result will be taken as the basic data for further evaluation. Additionally, at each color difference threshold, the traditional seed filling algorithm is independently executed based on the seed point of each zone. Secondly, the single seed filling results at each threshold are systematically compared with the multisource seed filling ones, especially in those key image regions of color transitions and blurred spatial features. Thirdly, the boundaries of the target water surface are obtained by performing contour detection on the results of multisource seed filling at each threshold. By comparing the characteristics of these boundaries, it can be clearly observed how different thresholds affect the accuracy of extracting the target water surface boundary. Finally, the optimal color difference threshold is determined according to the degree of geometric consistency between the extracted water surface boundary and the actual one. At the same time, the resulting boundary at the selected optimal threshold should avoid abnormal situations such as boundary breakage, incompleteness, or overflowing.

### 3.2. Affine Transformation of Normal Water Surface

After the boundary is accurately extracted, the image portion of the intact water surface in the UAV-acquired single image is conveniently clipped out. However, the clipped image cannot be directly used to restore the abnormal water surface in the DOM as they do not coincide with each other in orientation and scaling. Therefore, the clipped image should be transformed into the replacement image using a affine plane coordinate transformation [21,22], and the transformation parameters are calculated based on tie points manually selected from the water boundaries of the single image and the DOM. The formulas for the coordinate transformation [23,24] are as follows.

(2) $$x^{\prime }=a_{1}x+a_{2}y+b_{1}\;y^{\prime }=a_{3}x+a_{4}y+b_{2}$$

where (*x*, *y*) are the coordinates in the original image and (*x*′, *y*′) are the corresponding coordinates in the target image. $a_{1}$, $a_{2}$, $a_{3}$, $a_{4},b_{1}$, and $b_{2}$ are the parameters to be determined.

Equation (2) can also be expressed as the following matrix form.

(3) $$\left[\begin{array}{c} x^{\prime }\\ y^{\prime } \end{array}\right]=\left[\begin{array}{c} \begin{array}{ccc} a_{1} & a_{2} & b_{1} \end{array}\\ \begin{array}{ccc} a_{3} & a_{4} & b_{2} \end{array} \end{array}\right]\left[\begin{array}{c} x\\ y \end{array}\right]$$

**Figure 6:**
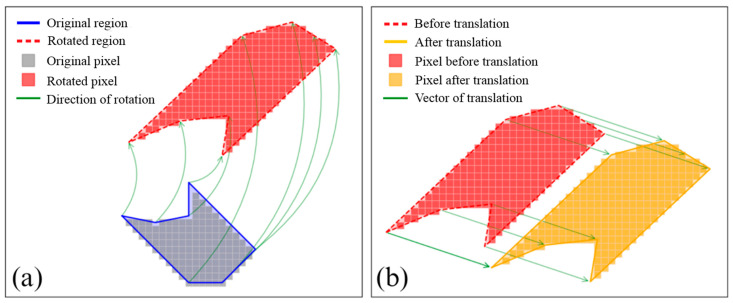
Affine plane coordinate transformation. (**a**) Rotation transformation; (**b**) translation transformation.

$a_{1}$, $a_{2}$, $a_{3}$, and $a_{4}$ constitute the rotation transformation matrix for handling the rotation, scaling, and shearing of the original image (Figure 6a), while $b_{1}$ and $b_{2}$ are the two elements of the translation vector for translating the rotated image (Figure 6b).

Therefore, to obtain the final transformation formula, it is actually necessary to determine the values of all elements in the 2 × 3 matrix above. This is usually performed by selecting at least three pairs of tie points in the original image and the target image. Then, each set of pixel coordinates (*x*, *y*) in the clipped image can be transformed into the coordinates (*x*′, *y*′) in the DOM, resulting in a replacement image.

The process of restoring abnormal water surfaces in the DOM is actually relatively simple, and involves a point by point replacement of the pixel values in the corresponding positions in the DOM with those in the replacement image.

## 4. Results

### 4.1. Extracted Normal Water Surface

#### 4.1.1. Selected Multisource Seed Points

**Figure 7:**
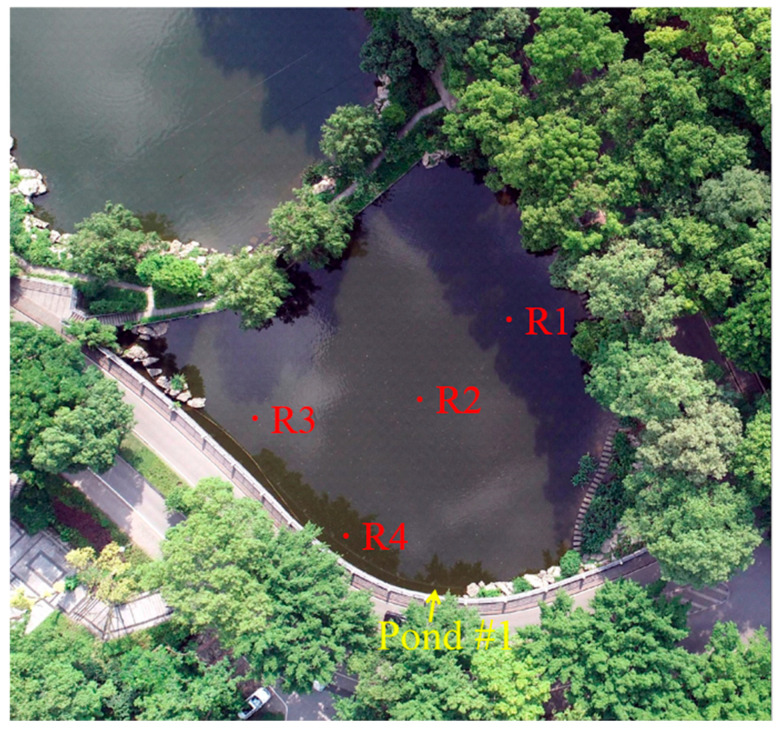
Distribution of selected seed points (R1, R2, R3, and R4) in the four color zones.

The first step of the multisource seed filling algorithm is to select the seed points according to the actual zoning of the selected single image containing the normal water surface. In addition, it is recommended that the selected seed points are located in the middle of the zones and not near the edges. As analyzed in Section 3.1.1, there were basically four color zones in Pond #1. Therefore, to ensure the complete coverage of the water surface area, the four fixed seed points were separately selected in the four zones (See Figure 7). Specifically, the seed point R1 was located in the shadow zone 1 (A) in the upper right of the image, responsible for identifying water areas shaded by near shore trees. The seed point R2 was positioned at the center of the dark green water zone (B), primarily identifying the open water area directly exposed to sunlight. The seed point R3 was located in the cloud reflection zone (C) in the upper left, focusing on the water area with cloud reflection. The seed point R4 was set in the lower-left shadow zone 2 (D) to identify strip-shaped shaded water area. Although the two zones (A and D) where R1 and R4 were located had similar spectral characteristics, the seed points were separately selected to ensure coverage of the two zones as their spatial positions were completely separated. Thus, it ensured that the filled results of the four seed points could fully cover the entire water surface, which is beneficial for determining a reasonable color difference threshold in subsequent steps.

#### 4.1.2. Determined Color Difference Threshold

After multiple tests, the color difference threshold selection fell into the interval of 6–18, and the candidate threshold sequence of 6, 12, and 18 was used for the following comparative analyses. Among them, the optimal threshold was selected for the execution of the multisource seed filling algorithm.

**Figure 8:**
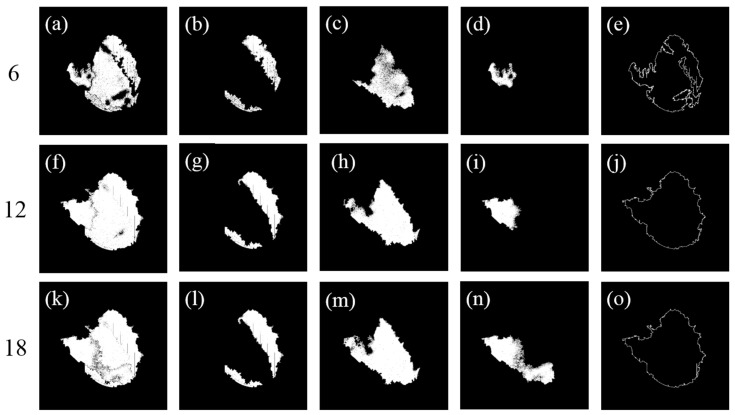
Overall filled water surface (**a**), filled shadow zones (**b**), filled dark green zone (**c**), filled cloud reflection zone (**d**), and extracted boundary of the entire water surface (**e**) at a color difference threshold of 6; overall filled water surface (**f**), filled shadow zones (**g**), filled dark green zone (**h**), filled cloud reflection zone (**i**), and extracted boundary of the entire water surface (**j**) at a color difference threshold of 12; and overall filled water surface (**k**), filled shadow zones (**l**), filled dark green zone (**m**), filled cloud reflection zone (**n**), and extracted boundary of the entire water surface (**o**) at a color difference threshold of 18.

Starting from the four seed points (R1–R4), the multisource seed filling algorithm was first used to collaboratively fill the four zones to obtain the overall water surface for each threshold (Figure 8a,f,k). Subsequently, each zone was separately filled starting from its own seed point using the algorithm. The filled shadow zones are jointly shown in Figure 8b,g,l, the filled dark green zones in Figure 8c,h,m, and the filled cloud reflection zones in Figure 8d,i,n for the three thresholds, respectively. Finally, the contour-finding algorithm was employed to extract the boundaries (Figure 8e,j,o) of the overall filled water surface scopes (Figure 8a,f,k).

When the color difference threshold was set to six (Figure 8a–e), the boundaries of the overall water surface were jagged. The cloud reflection zones and dark green zones in the upper left of the water surface were not fully identified, resulting in large gaps between the two zones. Additionally, some cracks were observed at the boundary between the shadow zone and the dark green zone. These issues were probably caused by significant color gradient changes in the boundary areas, which led to the incorrect division of the same water surface into multiple zones by the algorithm. In contrast, when the threshold was set to 18 (Figure 8k–o), repeated zone identification was observed, such as in Figure 8m,n, where the dark green zone at the bottom of the pond was simultaneously filled by R2 and R3. Moreover, the excessively high threshold usually significantly reduced the computational efficiency.

When the threshold was set to 12, the optimal results were achieved (Figure 8f–j). The filled area of each zone was found to closely match the actual water color zones in the single UAV image, and the best completeness for the overall water surface was achieved. The repeated recognition of areas and misclassification of objects were effectively avoided. It was worth noting that at the optimal threshold, the filled results still contained some small area voids. These voids were primarily caused by brightness anomalies due to sunlight reflection in small areas and noise interference in limited areas. However, these microscopic defects did not affect the overall identification of water surface edges, and the continuous water surface boundary (Figure 8j) was still accurately extracted using the contour-finding algorithm.

By comparatively analyzing the collaboratively and individually filled results at different color difference thresholds, it was demonstrated that the single seed filling algorithm could only separately achieve the recognition of each zone, whereas the proposed multisource seed filling algorithm was capable of handling the recognition of multiple discrete zones simultaneously. According to the combined result, the multisource seed filling algorithm was able to not only accurately identify all color zones, but also maintain a natural transition between color zones when dealing with complex zone edges. This proved the necessity and superiority of the multisource collaboration mechanism in addressing complex color-zoning tasks.

**Figure 9:**
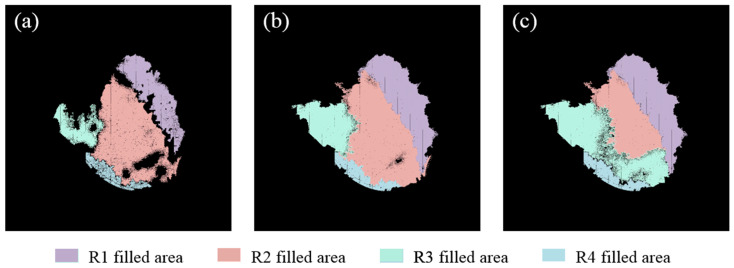
The color-rendered filled results using the multisource seed filling algorithm at color difference thresholds of 6 (**a**), 12 (**b**), and 18 (**c**).

In order to illustrate the role of each seed point in the multisource collaboration mechanism in a more intuitive way, the filled areas of different seed points were rendered using different colors. When the color difference threshold was set to 18 (Figure 9c), the filled areas of seed points R2 and R3 were significantly different from the results of the single seed filling algorithm (Figure 8m,n), which was manifested by the fact that the filled area of R2 over-expanded and encroached on the area that should be handled by R3. This led to the inability to fully reflect the filling effect of R3, or raised the issue of how to allocate overlapping areas between adjacent seed points. In contrast, when the color difference thresholds were set to 6 and 12 (Figure 9a,b), the shapes and scopes of the filled areas of each seed point were highly consistent with the results (Figure 8b–d,g–i) acquired by the single seed filling algorithm. This reflected the labor division and collaboration mechanism among the seed points. In addition, the colorful zoning results clearly demonstrated the unique advantage of the multisource seed filling algorithm with collaboration mechanism, namely the seed points shared the global traversal records during the synchronous filling processes. When the edges of the adjacent filling areas met, a natural fusion process was realized. This effectively avoided the phenomena of breakage and omission in the water surface and ensured the completeness and continuity of the filled results.

### 4.2. Restored DOM

**Figure 10:**
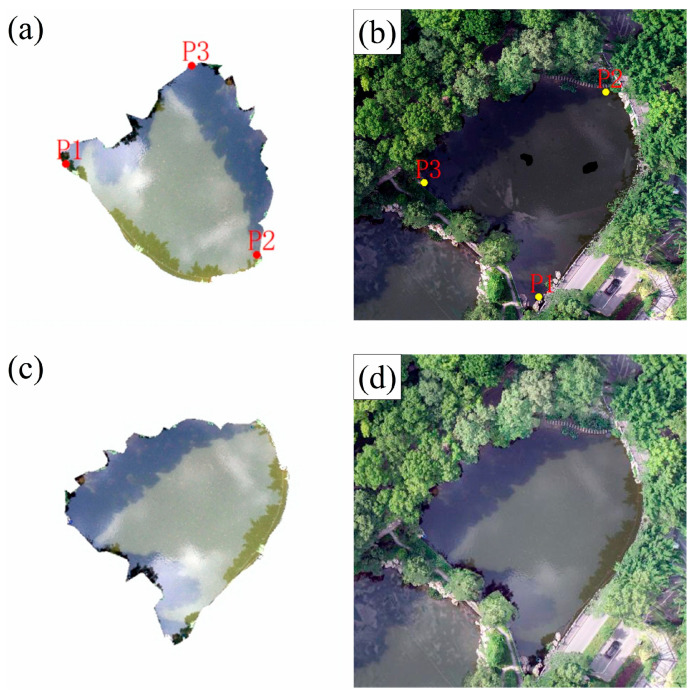
(**a**) Selected tie points (P1, P2, P3) on water edges of the extracted normal Pond #1; (**b**) selected tie points (P1, P2, P3) on water edges of Pond #1 in the DOM; (**c**) replacement image after affine plane coordinate transformation; and (**d**) restored DOM.

With the extracted water surface boundary at a color difference threshold of 12 (Figure 8j), the intact water surface of Pond #1 (Figure 10a) was clipped out from the single UAV image (Figure 1b). In affine plane coordinate transformation, at least three pairs of tie points were required to establish the mapping relationship between the extracted image and the DOM. To ensure the precise alignment of the water areas of Pond #1 in the two images, the selected tie points should have easily identifiable geometric features. As shown in Figure 10a,c, tie points P1, P2, and P3 were selected on the water edges of the extracted image and the original DOM, respectively. Then, the (*x*, *y*) coordinates of the three tie points were substituted into Equation (2) to obtain six equations, which were solved to obtain the six parameter values. The matrix form of the six parameter values was as follows:

$$\left[\begin{array}{c} \begin{array}{ccc} -0.1290990314060845 & 1.048253443219511 & 867.3564979545428 \end{array}\\ \begin{array}{ccc} -1.038523109625112 & -0.1243229847905974 & 2292.500151073617 \end{array} \end{array}\right]$$

Subsequently, the (*x*, *y*) coordinates of each pixel in the normal water surface image (Figure 10a) were transformed using Equation (2) into the (*x*′, *y*′) coordinates in the DOM (Figure 10c, namely replacement image). Then, the replacement image was overlaid on the DOM, and the pixel values of the replacement image replaced those at the corresponding positions in the DOM. Finally, the restored DOM was achieved (Figure 10d).

By comparing and analyzing the qualities of the DOMs before and after the restoration, it was found that obvious texture stretching and data holes in Pond #1 of the original DOM (Figure 1b and Figure 10b) were effectively eliminated. The restored DOM (Figure 10d) not only achieved the integrity of the image content, but also exhibited significant improvement in color consistency and texture continuity in the area of Pond #1, thereby realizing the expected restoration effect.

### 4.3. Quantitative Assessment

**Figure 11:**
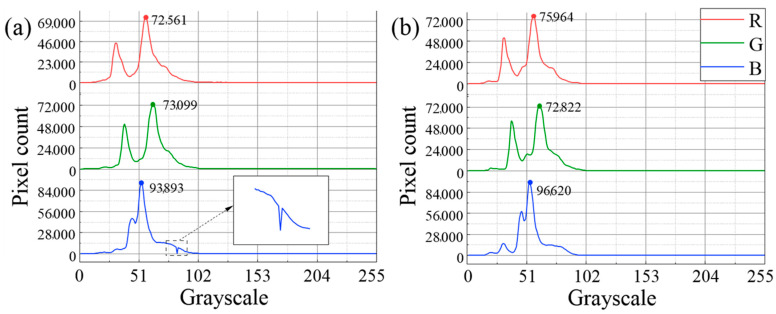
(**a**) Statistics of RGB pixel counts of the water surface area in the original DOM; (**b**) statistics of RGB pixel counts of the water surface area in the restored DOM.

**Figure 12:**
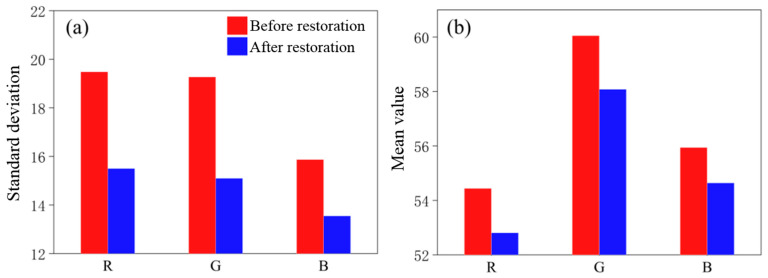
(**a**) Standard deviations before and after restoration; (**b**) RGB mean values before and after restoration.

In order to quantitatively assess the water surface restoration effect, a statistical analysis was conducted in this study from the following three aspects:

(1) Histogram analysis based on the distribution of band grayscale values [25]. Before the restoration (Figure 11a), the water surface area exhibited a more dispersed distribution of grayscale values in the blue, green, and red bands due to the texture stretching and data holes. There was an obvious abnormal valley in the blue band near the grayscale value of 80, and the count of pixels with the grayscale value of 52 was 93,893. After the restoration (Figure 11b), the distribution of grayscale values in each band became significantly concentrated. The count of pixels with the grayscale value of 52 in the blue band increased to 96,620, and the abnormal valley disappeared, fully reflecting the color homogeneity of the water surface [26].
(2) The standard deviation of band grayscale values. As indicated in Figure 12a, the standard deviations of the grayscale values of the three bands were significantly reduced after the restoration, confirming that the spectral consistency of the restored water surface had been notably improved. The color difference was reduced and color homogeneity was enhanced.
(3) The mean value of band grayscale values. As shown in Figure 12b, the highlight overexposure caused by texture stretching in the original DOM led to abnormally high RGB mean values in the water surface area [27]. After restoration, the mean values of RGB bands were significantly reduced, reflecting a more realistic water surface reflectance.

## 5. Discussion

### 5.1. Applicable Scenarios

The restoration method proposed in this study has particular scenarios for application, which are focused mainly on the tasks of eliminating water surface anomalies in small-scale water bodies. Specifically, water bodies such as ponds and river segments, which can be fully covered by a single UAV-acquired image, are considered ideal application targets for this method. This is due to the relatively limited spatial coverage of a single UAV image, which is usually determined by the pixel resolution of the camera and the flight height of the UAV relative to the ground.

If the multi-image stitching method [28,29] is used to expand the spatial coverage of the UAV images with a normal water surface, this method can theoretically restore larger-scale abnormal water surfaces. Nevertheless, many problems are faced in practice. Multiple-image stitching involves spatial alignment between adjacent overlapping images, which requires precise stitching algorithms and additional computation resources. Even during the stitching process of these images themselves, similar water surface anomalies may occur. These inevitably introduce more uncertainties, which may affect the accuracy and stability of the restoration results.

### 5.2. Appropriate Single Image Selection

In order to maximize the consistency of the restoration result, it is recommended to prioritize selecting the single UAV image with weather conditions consistent with the DOM to be restored. Weather conditions, especially lighting conditions, have a significant effect on the color tone and brightness of the images. The final color tone and brightness of the DOM is a reflection of the overall lighting conditions of the UAV-acquired images. Due to the relatively short operation time of the UAV and the high overlap rate of continuously captured images, it is usually not difficult to find images with similar weather conditions to the DOM. During the photographing process, there may be an occasion when clouds briefly obscure the sun, causing changes in lighting and water reflection. If the single image containing the complete water surface is exactly captured in this time period, the preprocessing techniques such as histogram matching can be used to reduce lighting differences.

Small geometric distortion is another important criterion for selecting a single UAV image. The DOM that had been orthorectified is used [30] as a reference for conducting such a visual selection. The selected image generally contains the corresponding water surface with a boundary shape geometrically approximating that in the DOM. There is no specific quantitative standard for approximation degree, as subsequent affine plane coordinate transformation can achieve good matching by stretching and resampling the normal water surface clipped from the single image. The selected image with high geometric approximation can greatly reduce the impact of affine transformation on the visual effect and quality of the final replacement image.

### 5.3. Edge Effect and Smoothing

According to Figure 10d, the restored DOM achieved a natural transition with the surrounding environment. But in other practical applications, noticeable edge effects may appear between the restored water surface and the surrounding areas. This edge effect is manifested in the discontinuity between the restored water area and the original DOM in terms of color and texture, thus affecting the overall quality of the restoration. To address this problem, edge-smoothing algorithms [31] can be introduced to eliminate or alleviate the edge effect in future research.

The edge-smoothing algorithms can achieve natural transitions between the restored water surface and the surroundings in several ways. One way is to implement a gradual color transition at the edge of the restored water surface [32,33], i.e., the color of the edge area is progressively adjusted, so as to achieve a smooth transition with the color of the surrounding features and reduce visual inconsistency caused by abrupt color change. Another way is to use edge fusion techniques based on texture synthesis [34], which can generate textures that match the visual characteristics of the surrounding elements. The generated textures will be integrated into the edge of the restored area to improve spatial consistency. Additionally, adaptive filtering algorithms [35,36] can be applied to automatically adjust filtering parameters based on local spatial features, achieving more precise edge smoothing. Recently, smoothing methods that adaptively adjust to texture intensity and enforce edge consistency have shown effectiveness for natural edge fusion [37]. These measures offer potential directions for further improving water surface restoration quality in the DOM, and are expected to significantly enhance the seamless integration between the restored water area and the surroundings.

### 5.4. Other Limitations and Potential Improvements

In addition to the aforementioned aspects, the proposed method also has other limitations such as manual and subjective interventions in the procedures of water surface zoning, tie point selection, and so forth.

In the study, the water surface was roughly zoned according to the distribution of several principal colors and textures, including the original color and texture of water, shadows, cloud reflections, etc. Then, the seed point of each zone was manually selected in the middle of the corresponding zone. Hence, the zoning and selection process was inevitably influenced by subjective factors. In future studies, some segmentation algorithms [38] can be adopted to automatically segment the water surface into some zones based on its principal colors and textures.

In affine transformation, the tie points on the water surface boundaries of the clipped image and the DOM were manually selected, which inevitably caused slight mismatches. Furthermore, the mismatched tie points may lead to incorrect water surface coverage and unresolved areas in the final restored DOM. To reduce the mismatching, the points with distinctive geometric features such as inflecting or protruding points can be selected on the water surface boundaries as tie points. Additionally, some tie point matching algorithms [39] can be used to automatically extract tie points for precise affine plane coordinate transformation.

Overall, the proposed method for restoring water surface anomalies was based on traditional image analysis and processing. Currently, deep learning is increasingly being applied in water surface recognition and mapping [40,41,42], and it is expected to achieve better results in removing water surface anomalies. However, current research in this regard is mainly limited to water quality anomaly detection [43,44]. Therefore, as a method that combines automation and manual participation, the effectiveness of the proposed method has been proved with the practical example in the study.

## 6. Conclusions

Due to the lack of significant textural features, some anomalies such as texture stretching or data holes frequently occur in water areas of the DOM produced from UAV-acquired overlapping images. These anomalies seriously impact the visual quality and data integrity. In this study, an innovative method based on local image replacement was proposed to eliminate the water surface anomalies in the DOM. The principal conclusions we reached were as follows:

- The proposed multisource seed filling algorithm is superior to the single seed one via conducting synchronous filling processes in all color zones. The sharing of the global traversal records among all of the filling process is the key point for reasonably dealing with the border conflicts of adjacent filling zones.
- As one key parameter for the multisource seed filling algorithm, the optimal color difference threshold can only be determined from a series of candidate thresholds through repeated testing. Each test includes the procedures of multisource seed filling and boundary extraction, and the effect of the tested color difference threshold is judged based on the integrity and continuity of the filled area and extracted boundary of the entire water surface.
- After image replacement, the water surface anomalies in the DOM were effectively eliminated. According to the statistics of the standard deviations and mean values of RGB pixels, the quality of the restored DOM was greatly improved in comparison with the original one. The restored water area achieved the ideal effect in terms of color consistency and spatial continuity.

Although this study provided a feasible approach to restoring the anomalous water surface in the DOM of UAV remote sensing, there were several limitations that influenced its wider application, including the limited area of the water surface, relatively strict criteria for single image selection, untreated edge effects, excessive manual interventions, and so forth. Hence, it is still very challenging to eliminate water surface anomalies in the DOM product of UAV remote sensing. In future studies, edge-smoothing algorithms can be incorporated to alleviate the edge effects. The image segmentation and tie point matching algorithms can greatly improve the automation degree in water surface zoning and tie point selection. In addition, deep learning is expected to achieve better results in removing water surface anomalies.

## Data Availability

The data presented in this study are available on request from the corresponding author.
